# Cognitive and Functional Assessment of Psychosis Stratification Study (CoFAPSS): Rationale, Design, and Characteristics

**DOI:** 10.3389/fpsyt.2018.00662

**Published:** 2018-12-03

**Authors:** Scott R. Clark, K. Oliver Schubert, Andrew T. Olagunju, Ellen Alexandra Lyrtzis, Bernhard T. Baune

**Affiliations:** ^1^Discipline of Psychiatry, School of Medicine, The University of Adelaide Adelaide, SA, Australia; ^2^Department of Psychiatry University of Lagos, Lagos, Nigeria; ^3^Department of Psychiatry, Melbourne Medical School, The University of Melbourne Melbourne, VIC, Australia

**Keywords:** bipolar disorder, clinical stratification, cognition, depression, function, schizophrenia

## Abstract

Prediction of treatment response and illness trajectory in psychotic disorders including schizophrenia, bipolar affective disorder, schizoaffective disorder, and psychotic depression is difficult due to heterogeneity in presentation and outcome. Consequently, patients may receive prolonged ineffective treatments leading to functional decline, illness chronicity, and iatrogenic physical illness. One approach to addressing these problems is to stratify patients based on historical, clinical, and biological signatures. Such an approach has the potential to improve categorization resulting in better understanding of underlying mechanisms and earlier evidence-based treatment with reduced side effect burden. To investigate these multimodal signatures we developed the Cognitive and Functional Assessment of Psychosis Stratification Study (CoFAPSS) employing a prospective study design and a healthy control group comparison. The main aim of this study is to investigate cognitive, and biological “genomics” markers of psychotic illnesses that can be integrated with clinical data to improve prediction of risk and define functional trajectories. We also aim to identify biological “genomic” signatures underpinning variation in treatment response and adverse medical outcomes. The study commenced in June 2016, including patients with primary diagnosis of psychotic disorders including schizophrenia, bipolar affective disorder, schizoaffective disorder, and psychotic depression according to DSM-5 criteria. The assessment covers a wide range of participant history (life stressors, trauma, and family history), cognitive dimensions (social perception, memory and learning, attention, executive function, and general cognition), measures to assess psychosocial function and quality of life, psychotic symptom severity, clinical course of illness, and parameters for adverse medical outcome. Blood is collected for comprehensive genomic discovery analyses of biological (genomic, transcriptomic, proteomic, and cell-biologic) markers. The CoFAPSS is a novel approach that integrates clinical, cognitive and biological “genomic” markers to clarify clinico-pathological basis of risk, functional trajectories, disease stratification, treatment response, and adverse medical outcome. The CoFAPSS team welcomes collaborations with both national and international investigators.

## Introduction

Psychiatric illnesses presenting with psychotic symptoms, including schizophrenia, bipolar affective disorder, schizoaffective disorder, and psychotic depression are highly prevalent, affecting up to 5% of the population, and are leading contributors to disability-adjusted-life-years, and years-lost-to-disability globally (1, 2). For example, in Australia the 12-month prevalence of psychotic illness managed within public mental health services was recently estimated at 4.5/1,000 people. The majority of these cases meet diagnostic criteria for schizophrenia or schizoaffective disorder (3, 4). Up to one half of these patients have reported a suicide attempt in their lifetime, over 60% report only partial recovery or continuous chronic illness, 32% have a severe dysfunction in the quality of self-care, and 85% rely on a government pension as their main source of income (3). There are high rates of comorbid chronic medical conditions and in a large Australian sample over 50% met criteria for metabolic syndrome, 75% were overweight or obese, nearly half had raised cholesterol or triglycerides, and one third had raised fasting glucose (5). Life expectancy in those with schizophrenia is decreased in excess of 16 years largely due to cardiovascular diseases (6, 7).

From longitudinal epidemiologic studies, it is clear that illness trajectory and progression in psychosis, varies greatly between and within individuals. For example, only about 20% of young people diagnosed as being at Clinical High Risk (CHR) for developing a psychotic disorder actually develop a full-blown mental illness (8), while 20% of those experiencing a psychosis will only have a single episode (9). At the other end of the spectrum, current treatments for schizophrenia are ineffective in up to 25% of cases, classified as treatment resistant (TRS), and only 30–60% of these patients will respond to the unique, second-line antipsychotic clozapine (10). While clinical factors conferring increased vulnerability to adverse outcomes in psychotic disorders, such as a prolonged duration of untreated psychosis (9), or non-adherence to prescribed treatments (11), have been well documented, the biology underlying these findings is not well defined. Further, trans-diagnostic categorization of psychotic disorders can be difficult because they often exhibit epidemiological comorbidity and share symptoms suggesting etiologic overlap through shared heritability (12). This is substantiated by genome-wide association studies (GWAS) showing high genetic correlations among psychotic disorders and overlapping of common risk variants across traditional diagnostic boundaries (12–14). Mapping genetic underpinnings of common psychotic disorders using cross-diagnostic design may therefore help in informing the search for the biological pathways underlying their pathophysiology, improve diagnosis, and treatment (12).

Currently, diagnosis and treatment recommendations for mental disorders are solely based on clinical assessments and broad clinical guidelines (15), and it is impossible to predict illness course and treatment response for individual patient (16). As a result, many patients receive treatments over prolonged periods of time which are either ineffective, or carry an unfavorable risk to benefit ratio, leading to illness chronicity, functional decline, poor adherence, or iatrogenic physical illness. Diagnostic categories in psychiatry are heterogenous in terms of presenting symptoms, underlying biology, and longitudinal outcomes. Stratification models for major mental illnesses can help to improve the efficacy of outcome prediction and intervention (17). Stratification is increasingly used in general medicine to define prognosis and allow personalized treatment. The process requires a clinico-pathological description using genetic and/or endophenotypic measures, and stratification into risk or response groups through the integration of patient's clinical data with other information including cognitive, neurophysiology, and biological genomics (18). Stratification may improve knowledge of underlying disease mechanisms via the development of “bio-signatures” that can characterize, validate or redefine clinical diagnosis (8, 19, 20). In turn, stratification may lead to early-targeted treatment with better initial response, favorable risk-benefit ratio, and modification of individual's risk of disease progression through state-appropriate treatments. For example in Oncology, molecular methods have led to better diagnosis and individualized treatment for cancer patients. Response to the anti-cancer drug–trastuzumab, a monoclonal antibody that blocks cell proliferation signals, is associated with over expression of the HER2 protein (21). Similarly, prognosis and the selection of chemotherapy for chronic lymphocytic leukemia is dependent upon the presence of clinical risk factors, cell surface antigens, and specific mutations such as the deletion of 11q or 17p and mutations of TP53 (22).

In order to truly progress preventive and personalized clinical approaches in psychosis, we need to understand the underlying psychological and neurobiological mechanisms and biomarkers associated with specific illness and functional trajectories. Similarly, we need to understand factors that promote functional regeneration and recovery following first episode psychosis. Recent years have seen progress in the identification of neurobiologically distinct biotypes developed using biomarkers across genetics, proteomics, neuroimaging, cognition, and electrophysiology leading to alternative classifications of psychotic illness (14, 23), improved prediction of transition to first-episode psychosis (24), response to lithium therapy in bipolar disorders (25, 26), and response and relapse in schizophrenia (27). However, the majority of these investigations have been carried out in small samples without replication and there is a need for larger cohorts of psychosis patients with adequate clinical and biological phenotyping. Such cohorts, in addition to yielding improved information about individual risk profiles for illness progression, would also allow for systematic assessment of the factors promoting individual patient's potential for full functional recovery and adverse outcome. To generate a deeply phenotyped psychosis cohort for the determination of multimodal signatures we developed the Cognitive and Functional Assessment of Psychosis Stratification Study (CoFAPSS) employing a prospective study design and a healthy control group comparison. The main aim of this study is to investigate cognitive, and biological “genomics” markers of psychotic illnesses that can be integrated with clinical data to improve prediction of risk and define functional trajectories. We also aim to identify biological genomic signatures underpinning variation in treatment response and adverse medical outcomes.

## Methods

### Research Aims and Hypotheses

The overall aim is to develop clinical, cognitive, and biological “genomic” markers of the risk of progression, functional trajectories, and outcomes in psychotic disorders that can inform stratification. The study hypotheses and specific aims are:

*Hypothesis 1:* Participants with psychosis, compared to matched healthy controls are characterized by specific genomic, transcriptomic, proteomic, and cell-biological signatures, which differ across illness trajectories and between risks for illness progression. The aims are: (a) to sample DNA, RNA, and protein expression in participants with psychosis and healthy matched controls, and (b) to compare global expression profiles focusing on heterogeneous illness trajectory.
*Hypothesis 2:* There are specific genomic, transcriptomic, proteomic, and cell-biologic signatures correlating with poorer neuropsychological function in the cognitive domains of social perception, memory and learning, attention, executive function, and general cognition, and these markers reflect the increased risk for disease progression in psychotic people with impaired cognition. The aims are: (a) to assess neuropsychological performance in the cognitive domains of social perception, memory and learning, attention, executive function, and general cognition in participants with psychotic disorders and in healthy matched controls, and (b) to correlate neurocognitive profiles with genomic, transcriptomic, proteomic, and cell-biologic signatures and the risk for disease progression.
*Hypothesis 3:* There are specific genomic, transcriptomic, proteomic, and cell-biologic signatures of favorable and unfavorable functional outcomes in people with psychosis. These markers may be similar or different to biomarkers correlating with cognitive impairment (see H2), and are specific to the various trajectories of psychotic disorders. The aims are: (a) to assess the functional outcome profiles of psychotic disorders, (b) to assess the relationship between functional outcome and biomarker signatures in psychosis, (c) to assess the relationship between cognitive function and functional outcome, and to compare their respective biomarker signatures, and (d) to assess the differences between subjective and objective rating of psychosocial functioning in people with psychotic disorders, and to correlate these differences to actual functional outcomes.
*Hypothesis 4:* There are specific genomic, transcriptomic, proteomic, and cell-biologic signatures of adverse medical outcomes in people with psychosis that are specific to psychotic disorders. The aims are: (a) to investigate metabolic, cardiovascular, hematological, gastrointestinal neurological outcomes in people with psychotic disorders, and (b) to investigate associations of these outcomes with genomic, transcriptomic, proteomic, and cell-biologic signatures.

### Study Design and Recruitment

The CoFAPS-Study employs methodology and instruments adapted in part from the Cognitive Function and Mood Study [CoFAM-Study] (28). The outlined CoFAPS-Study commenced in June 2016 with data collection at baseline, follow up assessment at 6-months and then annually for 3 years. The study design is prospective in nature and involves naturalistic recruitment of patients aged 18–65 years from inpatient and outpatient psychiatric services through research clinics of the Department of Psychiatry University of Adelaide, Central, Eastern, Western, Northern Adelaide, and Country Health Networks, South Australia. Healthy controls and people with a history of psychotic illness are also recruited from the general community via public advertisement. The study is exploratory in nature and forms the basis for biobanking of deeply phenotyped genomic and proteomic samples from patients with psychosis and healthy controls. *Post-hoc* power analyses will be performed to estimate achieved statistical power on the final data set.

### Inclusion and Exclusion Criteria

Participants with a current or previous diagnosis of psychotic disorders (e.g., schizophrenia, schizophreniform disorder, schizoaffective disorder, bipolar disorders, and psychotic depression) according to the Diagnostic and Statistical Manual of mental disorders- fifth edition-DSM-5 (29) are included in the study. We excluded potential participants unable to understand English, or to give informed consent, or tolerate assessment procedures. Those with impaired cognitive and functioning abilities associated with severe physical illness, comorbid developmental or neurological disorders, or learning disability are also excluded from the study. In addition, acutely distressed participants who display clear acute impairments of mental state requiring urgent medical or psychiatric attention are excluded. Healthy controls are expected not suffer from any disorder as defined by DSM-5.

### Ethics

The study was approved by Human Research Ethics at the Royal Adelaide Hospital (approval number: R20140709 HREC/13/RAH/281). Participants are provided all the study details in writing and in person before informed consent is obtained. Special care is taken in consent of those with impaired capacity or other vulnerability by involving parents, next of kin or legal guardian according to the principles of the Declaration of Helsinki.

### Clinical, Self-Report, and Cognitive Assessments

#### Diagnostic Screening and Interview

All participants with clinical diagnosis of psychotic disorders (including schizophrenia, schizoaffective disorder, bipolar affective disorder, and psychotic depression) made by their psychiatrists are screened for lifetime prevalence of mental illness including psychosis based on DSM-5 criteria (29). Specific scales are used to measure symptoms of psychosis, depression, anxiety, suicidality, psychosocial functioning, and health service use.

#### Demographics, Psychiatric, and Medical History

Basic demographics including age, gender, ethnicity, income, living circumstances, marital status are collected. A psychiatric history checklist is administered to capture key items including: age of illness onset, number of life-time episodes, number of hospitalizations, class of psychotropic medications used, and family history of mental illness. A physical illness checklist is administered to record any diagnoses of physical illnesses (including neurological disease, heart disease, diabetes, cancer) in participants, their parents, siblings, children, grandparents, uncle, aunt, spouse, and close relatives and cause of death in any close relatives.

#### Psychotic and Mood Symptom Severity, and Other Clinical Characteristics

The severity of psychotic symptoms is assessed using the Positive and Negative Syndrome Scale (PANSS) (30). The PANSS is a 30-item instrument designed to provide symptom severity across three subscales, namely positive (7-item), negative (7-item), and general psychopathology (16-item) scales. It is standardized, valid and sensitive to provide a balanced assessment of psychotic symptoms. To assess severity of depression and anxiety symptoms, the Structured Interview Guide of the Hamilton Anxiety and Depression Scale (SIGH-AD) (31) is also administered. The SIGH–AD is a 31-item structured interview that combines the Hamilton Depression Scale (HAM-D, 17 items) and the Hamilton Anxiety Scale (HAM-A, 14 items). Values over 15 represent clinically significant levels of anxiety or depression. In addition, overall symptom severity is rated using the Clinical Global Impression Severity Scale (CGI-S) (32). The CGI-S is frequently used in clinical research because of its face validity and practicability.

Suicidal ideation and behavior is assessed using the Colombia Suicide Severity Rating Scale (C-SSRS) structured interview. The C-SSRS measures 4 constructs of suicidality: severity, intensity, behavior, and lethality. The measure shows good divergent and predictive validity, high sensitivity, specificity, and is sensitive to change over time (33).

Extrapyramidal side effects are formally assessed using the Abnormal Involuntary Movement Scale (AIMS) (34) and the Barnes Akathisia Scale (35).

### Functioning

Participants are administered a range of functional assessments including the Functioning Assessment Short Test (FAST), the Specific Level of Functioning (SLOF) scale, and the Global assessment of Function Scale (GAF) and an employment related function questionnaire. The FAST scale consists of 24 items developed for the clinical evaluation of the main difficulties in daily functioning for psychiatric patients (36, 37). It is brief, easy to apply, and is available in several languages. The items are rated 0 (no impairment), 1 (mild impairment), 2 (moderate impairment), or 3 (severe impairment). Total FAST score ranges from 0 to 72. Higher scores indicate greater disability and scores above 11 indicate the presence of significant disability. The time frame for evaluation is the previous 14 days (36, 37).

The SLOF scale (38) includes participant ratings of their ability to perform 43 specific tasks encompassing 6 domains: (a) physical functioning (e.g., vision, hearing, and walking), (b) personal care skills (e.g., eating, personal hygiene, and dressing), (c) interpersonal relationships (e.g., forming and maintaining friendships, initiating contact with others), (d) social acceptability (e.g., verbally or physically abusing others, performing repetitive behaviors), (e) activities (e.g., shopping, self- medication, handling personal finances using a telephone), and (f) work skills (e.g., has employable skills, works with minimal supervision). Ratings are made on a 5-point Likert scale indicating the level of assistance the participant needs to perform the task, with higher score indicating better functioning. The SLOF has excellent reliability and validity (38) and is commonly used to assess functioning in patients with schizophrenia (39–41).

The GAF combines an evaluation of symptoms as well as relational, social, and occupational functioning on a single axis. The scale runs from 1 to 100 and is divided into 10 equal parts providing defining characteristics, both symptoms, and functioning, for each 10-point interval. A low rating reflects worse symptoms and a poorer level of functioning, whereas a high rating reflects less symptoms and a better level of functioning (42).

A self-made employment questionnaire is administered to the participants. This questionnaire was developed to assess the impact of cognitive problems on employment status and work productivity in individuals suffering from mood disorders (28). The questionnaire is an interviewer-administered instrument. The studied time frame refers to the current employment status of the participant in the first section and their work productivity over the last 7 days in the second section. It is quick and easy to administer (on average it takes approximately 5 min) and provides a comprehensive assessment of the impact psychosis-related cognitive dysfunction has on occupational functioning.

### Treatment Response to Psychotropic Medications

The Lifetime Psychotropic Treatment Response scale (LPTR) was modified from the Lithium Lifetime Treatment Response scale (LLTR) (43). The LPTR scale covers treatment response to various types of psychotropic medication and hence replaces the LLTR. Criterion A is used to estimate response to a specific treatment whilst Criterion B is used to establish whether there is a causal relationship between clinical improvement and the treatment. The ALTR scale is quick and easy to administer (on average it takes about 5 min) and gives an overall picture of the effectiveness of psychotropic medications.

### Collateral For Objective Functional Assessment and Confirmation of History

Consent is sought from each participant to contact a nominated member of their clinical treatment team who knows them well (e.g., their care coordinator or treating doctor) to assess insight, provide objective assessment of everyday functioning to confirm treatment and psychiatric history. Confirmation of history and insight is particularly important for participants with chronic psychosis whose self-report may be incomplete or unreliable (43). If the participant does not wish for a clinician to be contacted for this purpose, they can nominate a family member or friend, or can indicate that nobody should be contacted.

### Self-Report of Additional Clinical Characteristics

Participants complete a self-report battery using standardized scales designed to assess perception of stress, coping strategies, health beliefs, general capacity to function, health service utilization, and quality of life. All components are derived from well validated and widely used measures that are available in the public domain.

The Family Inventory of Life Events (FILE) provides an index of 71 family stressors occurring during or prior to the last 12 months (44). The Perceived Stress Scale (PSS) contains 14 items that assess how often a participant felt under stress, rated on a 5 point likert scale over the preceeding month (45–48). The Short Form Health Survey (SF-36) is a set of 36 quality of life measures that can be scored in specific functional domains (49–53). The Childhood Trauma Questionnaire (CTQ) is a set of 28 items that explore positive and negative experiences during childhood including neglect and abuse (54). The Resilience Scale is a 26 item measure of coping ability and positive perspective, each rated on a 7 point likert scale Scale (55). The Health Beliefs Questionnaire is a 20-item scale assessing an individual's beliefs about their personal health, relationships and life course, each rated on a−5 to +5 likert scale. The health service utilization questionnaire is a locally developed measure of the participant's interaction with health care systems across medical and allied health (28).

### Cognitive Assessments

Participants are administered a series of paper and computer-based game-like activities designed to assess memory and learning, attention and working memory, social cognition, and executive function (28, 56, 57). All tests have been psychometrically validated and are used extensively in cognitive function research. The Psychology Experiment Building Language (PEBL) is free, open-sourced software that allows design sharing, and modification of ~70 behavioral tests (58) We chose this battery because it is robust, available on a range of platforms, and offers a range of cognitive tests that are appropriate for our study objectives. The CoFAPS-Study uses the Tower of London and the Stroop Color Word Test (SCWT) from the PEBL. The Tower of London Test (59, 60) is widely used for the assessment of executive functioning, specifically to detect deficits in planning that may occur in a variety of medical and neuropsychiatric conditions. The test requires the participant to shift a series of discs one at time across 3 pegs to match a suggested pattern. The SCWT measures processing speed, attention, cognitive flexibility and working memory (61–63). The test utilizes the “Stroop Effect,” which is the cognitive interference that occurs when the processing of a specific stimulus feature (e.g., word meaning) impedes the simultaneous processing of a second stimulus attribute (word color). Participants are requested to respond to the color of the word and not its meaning which is also a color. The administration of this test takes 7–10 min.

The Repeatable Battery for the Assessment of Neuropsychological Status (RBANS) is a brief paper-based assessment of neurocognitive status. The RBANS is validated for people aged 12–89 years, as a screen for cognitive decline or abnormal functioning (64) and has also been validated in studies of psychotic illness, depression, and dementia (65). The battery gives scaled index scores for five cognitive domains including immediate memory, visuo-spatial/constructional, language, attention, and delayed memory. The instrument has been shown to have reliability, test-retest stability, construct validity, inter-rater reliability, and content validity (56, 57).

The THINC tool battery is a brief screening instrument designed to detect cognitive deficits by employing a variety of well-established cognitive tests in a gamified platform (66). The tool contains tests of digit symbol substitution test (67), the choice reaction time test (68), the trail making test B (69), the n-back working memory paradigm (70), and a self-report 5-item questionnaire on perception of cognitive function—the perceived deficit questionnaire (PDQ-5) (71). It has been validated for use in major depression (72). The digit symbol substitution test is a measure of attention, perceptual speed, motor speed, visual scanning, and memory (67). The test requires the examinee to identify a unique geometric shape with its corresponding number provided in a key containing numbers one to six. The task of the participant is to match the number with the corresponding symbol when a series of number is shown on the screen. The Choice Reaction Time test measures both psychomotor speed and choice reaction time (68) Participants are asked to respond by pressing arrow keys pointing to the right or left side of the keyboard corresponding to the direction an arrow on the screen is pointing. The N-Back test measures executive control of information updating in working memory (70). In this test, participants are presented with series of symbols moving at a constant rate. The task is to map a target symbol to the one they have seen recently (one position back) that is hidden and press the correctly corresponding letter key. The Trail-Making Test B measures visual attention, visual speed, search speed, scanning, mental flexibility, processing speed, and executive function (69). Participants are expected to connect a set of 18 dots as fast as possible while still maintaining accuracy. The dots include both numbers and letters in ascending order, and participant draws lines to connect the dots in an ascending pattern, with the added task of alternating between the numbers and letters (i.e., 1-A-2-B-3-C, etc.). The PDQ-5 is a 5 item self-report questionnaire asking participants to rate problems with memory, attention or concentration over the previous 7 days, on a 5 point likert scale (71).

Social cognition is assessed using components of the Wechsler Adult Intelligence Scale Advanced Clinical Solutions Package (WAIS-IV-ACS), a well validated and widely used battery (73). The WAIS-ACS provides an integrated test of interpretation of facial affect, prosody, body language, and mental state interpretation. Assessors follow a paper-based protocol in which the participants are shown a series of photographs of people and interpersonal interactions, displaying different emotions, and behavioral scenarios. Participants are asked to interpret these emotions based on the photographs alone and in the context of recorded speech designed to simulate more nuanced emotional expression such as sarcasm. We use the three subtests, facial affect naming, prosody-face-matching, and prosody-pair-matching to test different aspects of social cognition (56, 57).

### Physiological Measures

A wide range of physiologic measures are collected including body temperature in degrees Celsius, weight in Kilograms, height in meters, blood pressure in millimeters of mercury, heart rate per minute, and blood sugar level in milli-moles per liter. A peripheral blood sample is taken at the time of assessment by a person qualified for venepuncture. Samples are processed to provide storable DNA, RNA, serum, and blood cell specimen in line with standard operating procedures. The storage of biomaterials is split between refrigerators, all of which are monitored by a central alert system continuously 24 h/day 7 days/week.

Participant DNA and RNA will be extracted from whole blood samples. Blood proteins will be derived from whole blood, serum, and plasma samples. Genetic variation amongst participants will be assessed by DNA microarrays. These data will contribute to international consortium initiatives pursuing genome-wide association studies (GWAS) in the field of psychosis research (74); additionally, genetic data will be used to determine the role of polygenic scores (PGS) for psychiatric and somatic phenotypes in the trajectory differentiation of psychotic disorders (25, 75). Differences in gene expression between better and poorer illness trajectories will be undertaken using RNA sequencing (76). We will employ “classic” differential expression analysis as well as systems biology approaches including weighted gene co-expression network analysis (WGCNA) (77) and analysis of expression quantitative trait loci (eQTL) (78), which we have previously successfully used in complex psychiatric traits (79–81). For proteomic analyses, we will use liquid chromatography-tandem mass spectrometry (LC-MS/MS) technology to perform: (1) differential expression analysis in shotgun discovery experiments, (2) semi-targeted analyses using data independent acquisition (DIA) approaches (82), and (3) targeted testing for promising markers using multiple reaction monitoring (MRM) (83).

### Quality Assurance and Data Management

The main purpose of all quality assurance processes is to derive high-quality data. The CoFAPS-Study standard operation manual contains operating procedures for recruitment, clinical interviews, physical examination, blood collection and storage, and handling of bio-specimen, Human Biobank and Genetic Research Database. Members of CoFAPS-Study team have training before commencement of study and follow-up quality checks are carried out. Performance is closely supervised, monitored, and routinely reviewed to ensure adherence. Data collection and management were implemented concurrently based on standardized procedures, and partly automated procedures for data processing and credibility checking. Data backup routines are scheduled on a daily basis.

### Biometric Concept and Statistical Analyses

The primary endpoints of CoFAPSS are the detailed characterization of trajectories of symptoms, cognition, psychosocial and general function, and associated genomic, protein, lipid and metabolomic markers of psychotic illnesses derived from peripheral blood. Secondary outcomes include changes in these variables over time. Depending on the type of outcome scale (continuous vs. categorical), and time point of assessment (baseline or follow-up), the statistical methods comprise of multivariable linear regression or logistic regression analyses or mixed-models and latent class or growth mixture modeling to identify predictor and outcome trajectories (84), accounting for time-varying predictors and repeated outcome assessment. Specific software is required for genetic, gene expression network and proteomic analyses.

Multimodal data will be integrated into personalized prediction models using a range of machine learning techniques including naive Bayes' (85, 86), penalized regression, support vector machines, random forest and artificial neural networks (87). To avoid data leakage and model overfitting, all models will be implemented within in a pipeline architecture using either Scikit-learn (88) or the Caret package in R (89) with k-fold cross validation including all data pre-processing, feature selection and classification processes. Model performance will be described in terms of variance explained (*R*^2^) in regression, or for classification discriminative ability (sensitivity and specificity), positive and negative predicted value, area under the receiver operating curve (ROC-AUC), F1, and goodness-of-fit statistics for calibration (90).

### Study Implementation and Dissemination

To date, data has been collected from 74 patients with established psychosis (75% schizophrenia, 25% schizoaffective disorder) recruited from inpatient and outpatient psychiatric services through research clinics of the Department of Psychiatry University of Adelaide, Central, Eastern, Western, Northern Adelaide, and Country Health Networks, South Australia. Participants are mostly male (70%) caucasians (86.3%), with average age 39.9 yrs (range 19–61), reflecting the local public outpatient clinic population. Data collection will continue as required to build robust multimodal models, with a focus on broadening recruitment to include controls, balance gender and include earlier age groups with first presentations of psychotic symptoms. We have presented baseline clinical, cognitive and functional data at National and International conferences (91, 92) and plan publications on baseline biological markers in international peer reviewed journals.

## Discussion

Psychiatric illnesses with psychotic features comprising of schizophrenia, bipolar affective disorder, schizoaffective disorder, and psychotic depression are heterogeneous disorders associated with chronic or recurrent disabling symptoms (1, 2). To address the limited diagnostic reliability, improve prediction of treatment response, reduce risk-benefit ratio, and personalize recovery-oriented care, a stratification of psychotic disorders is proposed (17–19). Such a stratification model requires multi-dimensional linkage of clinical, neurophysiological, and neurobiological factors to define illness trajectory and plan treatment. The CoFAPS-Study aims to improve the understanding of neurobiological and cognitive underpinnings of psychotic illnesses to advance classification, predict risk, and personalize treatment to promote recovery and prevent adverse outcome. Enrolment of cases and controls into CoFAPSS commenced in 2016 and will continue until December 2020.

Firstly, the CoFAPS-Study is designed to identify cognitive, functional, and biological “genomic” markers of psychotic illness trajectories in comparison to healthy controls. The main goal is to improve the characterization of psychosis and prediction of symptomatic and functional outcomes by incorporating neurobiological and psychosocial correlates. The design is similar to CoFAMS (28) for cross disorder comparison, and the data set is well characterized for consortium work and suitable for systems biology approach.

Secondly, we hope to improve understanding of the decline in psychosocial function and highly variable recovery in psychotic illness by exploring the validity of stratifying patients across different illness trajectories. Stratification of psychosis into functional trajectories will help to characterize those at early risk of long term poor outcomes.

Third, genomic, transcriptomic, proteomic, cell-biologic signatures, and neuropsychological correlates of treatment response and adverse medical outcome are specifically lacking in the field. A possible reason is the heterogeneous nature of psychosis as described in current syndromic classifications. The integration of illness and functional trajectories with predictive biological markers will assist in the early personalisation of care to optimize outcomes in psychotic illness.

In conclusion, the CoFAPS-Study is a novel approach utilizing clinical, cognitive, and biological “genomic” markers to improve prediction of risk, psychosocial function, and treatment response in psychotic disorders. The CoFAPSS team welcomes collaborations with both national and international investigators.

## Author Contributions

SC, KS, and BB conceived and designed the study. AO and EL provided knowledge on the execution of study and made substantial contributions to the acquisition of the data. AO and SC drafted the manuscript, all authors read and approved the final manuscript. BB provided supervision as the Lead Principal Investigator on the project.

### Conflict of Interest Statement

The authors declare that the research was conducted in the absence of any commercial or financial relationships that could be construed as a potential conflict of interest.
